# Effect of Hemi-Castration on the Productivity, Histological Characteristics, and Economic Efficacy of Korean Beef Cattle

**DOI:** 10.3390/ani11092490

**Published:** 2021-08-25

**Authors:** Jun-Sang Ahn, Eung-Gi Kwon, Hyun-Jeong Lee, Eun-Mi Lee, So-Mi Hwang, Sang-Rae Cho, Kyung-Woon Kim, Ui-Hyung Kim, Jeong-Il Won, Shil Jin, Sung-Sik Kang, Byung-Ki Park, Gi-Suk Jang, Sun-Sik Jang

**Affiliations:** 1 Hanwoo Research Institute, National Institute of Animal Science, RDA, Pyeongchang 25340, Korea; dkswns121@korea.kr (J.-S.A.); hyunj68@korea.kr (H.-J.L.); board11@korea.kr (E.-M.L.); somi171018@korea.kr (S.-M.H.); chosr@korea.kr (S.-R.C.); kw72kim@korea.kr (K.-W.K.); uhkim@korea.kr (U.-H.K.); won51@korea.kr (J.-I.W.); jins21@korea.kr (S.J.); sskang84@korea.kr (S.-S.K.); shinnanda16@naver.com (G.-S.J.); 2 Department of Animal Science, Kangwon National University, Chunchoen 24341, Korea; kug2237@kangwon.ac.kr (E.-G.K.); animalpark@kangwon.ac.kr (B.-K.P.)

**Keywords:** adipocytes, Hanwoo, hemi-castration, marbling score, net income, testosterone

## Abstract

**Simple Summary:**

Steers and bulls exhibit clear differences and corresponding disadvantages. We evaluated that the effect of hemi-castration on the growth performance, serum testosterone levels, carcass traits, histological characteristics, and economic efficacy of Korean beef cattle (Hanwoo) to determine if hemi-castration can compensate for the disadvantages of steers and bulls. Results show that hemi-castration achieve better average daily gain and carcass weight than castration, but it lowers the marbling score and auction prices; thereby negatively affecting net income. This is due to the constant release of testosterone from one testicle. Based on the findings of this study, castration is essential to produce high-quality beef because the level of serum testosterone secreted in hemi-castration can inhibit fat development as much as in bulls.

**Abstract:**

We evaluated the growth performance, serum testosterone, carcass traits, histological characteristics, and economic efficacy of castrated and hemi-castrated Korean beef cattle. Thirty-two Hanwoo calves (Initial body weight: 148.4 ± 19.8 kg) were randomly assigned into the castrated Hanwoo (CH) and hemi-castrated Hanwoo (HH) group. The experiment lasted 18 months; the animals were all slaughtered on the same day. Final body weight and average daily gain (ADG) tended to increase in the HH group compared to the CH group. Testosterone concentration was higher in HH group (5.27–14.27 ng/dL) than in the CH group (0.47–0.70 ng/dL) during the whole experimental period after castration (*p* < 0.05). Rib eye area was 17.08 cm^2^ wider in HH group than in CH group, but marbling score was improved by 3.33 in CH group compared to HH group (*p* < 0.01). Deposition area of adipocytes in *Longissimus dorsi* were higher in CH group than in HH group (*p* < 0.001). Net income per head was 1760 US dollar higher in the CH group than in the HH group (*p* < 0.04). Thus, our findings suggest that hemi-castration had positive effects on the increase in ADG and meat yield traits, with negative effects on marbling and profitability.

## 1. Introduction

Intramuscular fat (marbling) is the most important factor affecting carcass quality and price competitiveness in beef cattle. Castration is one of the most effective methods to increase intramuscular fat as it increases fat deposition by decreasing testosterone [1] which negatively affects the differentiation and proliferation of adipocytes [2]. In fact, a report by the Korea Institute for Animal Product Quality Evaluation (KIAPQE) [3] showed that the average marbling score of steers is 4.5 points higher than that of bulls, with a castration rate of approximately 97.6%. In addition, castration has the advantage of improving the tenderness of the meat and reducing aggressive behavior in bulls to facilitate management. Therefore, castration is an essential method for producing high-quality beef. Previous studies have analyzed the optimal methods and periods of castration for enhancing the castration effect [4,5]. However, due to decreased development of the urethra, castration also increases the probability of urinary calculi in steers [6] and reduces their average daily gain (ADG) and feed efficiency compared to those of bulls [7,8]. On the other hand, although bulls exhibit excellent ADG and carcass weight, the reduced formation of intramuscular fat makes them economically disadvantageous [9]. Thus, steers and bulls exhibit clear differences and corresponding disadvantages [10].

Since the most testosterone is produced by two testes, removal of one testicle may reduce testosterone levels, which has the potential to improve intramuscular fat accumulation, while maintaining proper growth. Thus, in this study, we hypothesize that hemi-castration (the removal of only one testicle) is the most efficient castration method to compensate for the respective disadvantages of steers and bulls. Specifically, we evaluate the hemi-castration effect by comparing the growth performance, serum testosterone levels, carcass traits, tissues characteristics, and economic efficacy of castrated and hemi-castrated Korean beef cattle.

## 2. Materials and Methods

### 2.1. Management and Use of Animals

Thirty-two Hanwoo calves (Initial body weight: 148.4 ± 19.8 kg, age: 5.9 ± 0.1 months) were used in this study. Calves were randomly assigned into the castrated Hanwoo (CH) group or hemi-castrated Hanwoo (HH) group, and surgically castrated at seven months of age. Four calves were allocated per pen (5 × 10 m) with sawdust to a thickness of approximately 30 cm. The growing period was 6–14 months of age, during which the animals were fed formula feed (3.0–7.5 kg) and Italian ryegrass hay (3.0–4.0 kg). The fattening period was 15–24 months of age, during which formula feed (8.0–9.5 kg) and rice straw (1.0–3.0 kg) were provided. Feed was provided twice a day, and water and mineral blocks were freely available. The dry matter, crude protein, ether extract, crude fiber, and crude ash contents of the experimental diets were analyzed using the AOAC [11] method, and neutral detergent fiber and acid detergent fiber were analyzed using the method described by Van Soest et al. [12]. The ingredient and chemical composition of the experimental diets are listed in Table 1.

### 2.2. Growth Performance

The ADG was calculated by measuring body weight (BW) at 09:00 every month. Dry matter intake (DMI) was calculated by measuring the quantity of residual feed per pen before feeding in the morning. The feed conversion ratio (FCR) was calculated based on DMI and ADG values.

### 2.3. Serum Metabolites and Testosterone Analysis

Blood collection was conducted at monthly intervals from the beginning to the end of the experiment. Before feeding in the morning, blood was collected (within 10 s) from the jugular veins of all animals using an 18-gauge needle and a 10 mL vacuum tube. Samples were centrifuged at 1250× *g* for 20 min. Serum was obtained by collecting the supernatant; 1 mL of each sample was divided into two microtubes and stored at −80 °C until analysis. Serum metabolites were analyzed using an automatic blood analyzer (Hitachi 7020, Hitachi Ltd., Tokyo, Japan), and the analysis items were glucose, cholesterol, triglyceride, and blood urea nitrogen (BUN). Testosterone levels were analyzed using the bovine testosterone ELISA kit (CSB-E13194B, CUSABIO, Houston, TX, USA). Fifty microliters of serum was added to each well of the microtiter plate (pre-coated with goat-anti-rabbit antibody), to which 50 μL of HRP-conjugate and antibody were added. The mixture was then incubated at 37 °C for 1 h. Each well was aspirated and washed three times using wash buffer (200 μL). Fifty microliters of substrate A and 50 μL of substrate B were added into each well, which were then incubated at 37 °C for 15 min. We then added 50 μL of Stop Solution to each well and determined the optical density of each well within 10 min, using a microplate reader set to 450 nm.

### 2.4. Carcass Traits

All animals were slaughtered at a local slaughterhouse. Carcass yield grades (carcass weight, back-fat thickness, rib eye area, and yield index) and quality grades (marbling score, meat color, fat color, texture, and maturity) were examined according to the criteria of the Korean carcass grading system [13]. The carcass was chilled for 24 h, and the weight of the cold carcass was measured. Next, the left side of each carcass was cut between the thirteenth rib and the first lumbar vertebra and used to determine the grade of yield and quality. The rib eye area was measured from the *longissimus* muscle at the thirteenth rib. Back-fat thickness was measured at the thirteenth rib. 

The yield index was calculated as follows:

Yield index = [(11.06398 − 1.25149 × back-fat thickness (mm)] + [0.28293 × rib eye area (cm^2^)] + [0.56781 × carcass weight (kg)]/[carcass weight (kg) ×100]

Yield grades were classified as grade A (best, yield index > 67.20), grade B (yield index 63.30–67.20), and grade C (worst, yield index < 63.30) as determined by the yield index. Yield grade scores was calculated as grade A = 3, grade B = 2, and grade C = 1. 

The quality grade was determined by assessing the degree of marbling in the cut surface of the rib eye based on the maturity, texture, meat color, and fat color of the carcass. The marbling scores were graded on a scale of 1 to 9, with higher numbers indicating better quality (1 = devoid, 9 = abundant). Additional scores included those for meat color (1 = bright red, 7 = dark red), fat color (1 = creamy white, 7 = yellowish), maturity (where 1 = youthful and 9 = old), and texture (where 1 = soft and 5 = firm). 

The quality grades were evaluated as follows: 1^++^ (excellent quality), 1^+^ 1, 2, and 3 (low quality). The quality grade scores were calculated as 1^++^ = 5, 1^+^ = 4, 1 = 3, 2 = 2, and 3 = 1. 

The auction price was calculated as the final successful bid price at the auction based on the quality grade quality and yield of carcass.

### 2.5. Histological Characteristics

After the carcass was evaluated, muscle tissue was collected from the *longissimus dorsi* (LD) and *semimembranosus* (SM), and tissue samples were fixed in fresh 10% (*w*/*v*) formalin in a phosphate buffer (pH = 7.4). The specimens were oriented for transverse fiber sectioning, dehydrated in a graded series of ethanol, and embedded in paraffin. From each sample, 10 serial transverse sections (6 μm thick) were obtained, mounted on poly l-lysine-coated slides, and stained with hematoxylin and eosin. The stained slides were observed with an optical microscope (Nikon Ni-U with DS-Ri2 Camera, Tokyo, Japan), and the characteristics of adipocytes and muscle fibers were analyzed using NIS-Elements BR Basic Research software.

### 2.6. Statistical Analysis

All data were analyzed by one-way analysis of variance (ANOVA) using the generalize linear model (GLM) procedure of SAS 9.3 (SAS Institute, Cary, NC, USA, 2012), with the addition of CH and HH as the only sources of variation. Growth performance, serum metabolites, and testosterone were analyzed as time-repeated measures. Within each replicate, triplicate samples were included for histological measurements. The results are presented as means and standard deviations; differences between treatments were considered significant at *p* < 0.05.

## 3. Results

### 3.1. Growth Performance

The growth performance of castrated and hemi-castrated Hanwoo are shown in Table 2. There was no difference in the initial BW between groups; however, the final BW tended to be higher in the HH group than in the CH group (*p* = 0.07), and the ADG showed similar results (*p* = 0.06). DMI was significantly higher in the HH group than in the CH group (*p* < 0.02), and the influence of formula feed intake was significant (*p* < 0.01). The FCR was 6.7% less in the HH group than in the CH group; however, this difference was not significant.

### 3.2. Serum Metabolites and Testosterone

**Figure 1:**
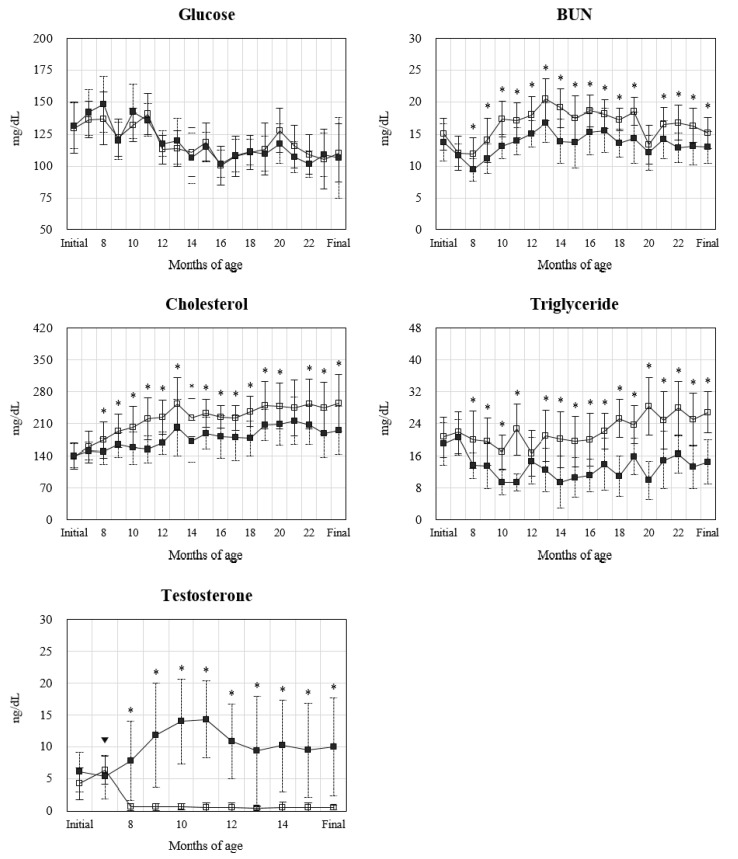
Concentration of serum metabolites and testosterone of castrated (CH: □) and hemicastrated (HH: ■) Hanwoo (* = *p* < 0.05; mean ± SD). Castration (▼) was performed at seven months of age. Serum testosterone levels were measured only up to 16 months of age.

The concentrations of serum metabolites and testosterone in castrated and hemi-castrated Hanwoo by age (months old) are shown in Figure 1. There was no difference in serum glucose concentrations between the treatment groups during the whole experimental period. However, serum BUN concentration was lower in the HH group than in the CH group from 8 to 24 months of age due to castration, except at 20 months of age (*p* < 0.05). In addition, serum cholesterol and triglyceride concentrations also differed between the treatments from 8 months of age in relation to castration and were mostly higher in the CH group than in the HH group (*p* < 0.05). The serum testosterone levels did not differ between the groups prior castration but was then higher in the HH group than in the CH group until 16 months of age according to castration (*p* < 0.05). In particular, the CH group exhibited a sharp decrease in testosterone concentration after castration to less than 0.70 ng/dL, whereas the HH group increased to a maximum of 14.27 ng/dL at 11 months of age before decreasing thereafter.

### 3.3. Carcass Traits 

The carcass traits of the castrated and hemi-castrated Hanwoo are shown in Table 3. There was no significant difference in carcass weight between the CH and HH groups. The rib eye area was wider in the HH group than in the CH group (*p* < 0.01), but the back fat thickness was much thicker in the CH group compared to the HH group (*p* < 0.01). Yield index was higher in HH group than in CH group due to the effects of rib eye area and back fat thickness (*p* < 0.01); however, no difference in yield grade score was observed between the treatment groups. Marbling score was significantly improved in CH group compared to HH group (*p* < 0.01), and meat color was darker in HH group than in CH group (*p* < 0.02). No effects of HH group were observed for fat color and maturity, but texture was higher in HH group than in CH group (*p* < 0.01). Quality grade score was more than twice as high in CH group than in HH group (*p* < 0.01), and auction price was 39.8% higher in CH group than in HH group due to the influence of marbling score (*p* < 0.01).

### 3.4. Histological Characteristics

**Figure 2:**
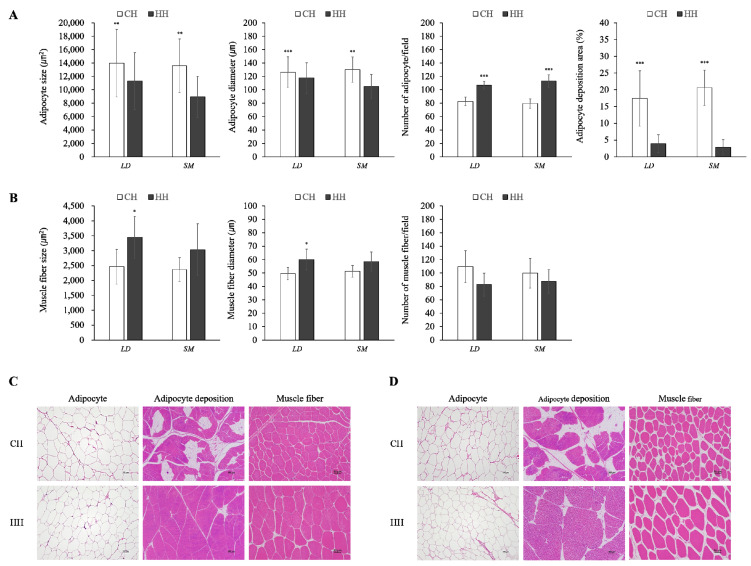
Characteristics of adipocytes (**A**) and muscle fibers (**B**) in skeletal muscle from castrated (CH: □) and hemi-castrated (HH: ■) Hanwoo (* *p* < 0.05; ** *p* < 0.01; *** *p* < 0.001; mean ± SD). (**C**,**D**) Representative images of adipocyte (100-fold), adipocyte deposition (20-fold), and muscle fiber (200-fold) stained with hematoxylin and eosin ((**C**) = LD, (**D**) = SM).

The characteristics of adipocytes and muscle fibers in the skeletal muscle of castrated and hemi-castrated Hanwoo are shown in Figure 2. Adipocyte size (*p* < 0.01) and diameter (LD: *p* < 0.001, SM: *p* < 0.01) were higher in the CH group than in the HH group. The number of adipocytes per field was higher in the HC group because of the larger adipocyte size in the CH group (*p* < 0.001). The adipocytes deposition area in skeletal muscle was significantly higher in the CH group than in the HH group (*p* < 0.001). The fiber size and diameter of muscle in LD were greater in the HH group than in the CH group (*p* < 0.05); however, no significant difference was observed in SM parameters between treatment groups or in the number of muscle fibers per field.

### 3.5. Economic Efficacy

Table 4 shows the economic efficacy of castrated and hemi-castrated Hanwoo. Owing to the high CH group auction price, the gross receipts rice was higher in the CH group than in the HH group (*p* < 0.04). Conversely, feed costs were higher in the HH group than in the CH group due to the effect of fattening formula feed (*p* < 0.01), and the roughage did not differ between the treatment groups. The net income was significantly higher in the CH group than in the HH group (*p* < 0.04).

## 4. Discussion

The results of this study suggest that hemi-castration may increase the growth and meat yield traits of beef cattle but may negatively affect the production and profitability of high-quality beef with high intramuscular fat due to the effect of testosterone secreted by one testis. Testosterone secreted from the interstitial cells of the testis maintains secondary sexual characteristics (SSC) and enhances protein synthesis [14]. In particular, testosterone promotes an increase in satellite cells and myonuclei [15] and affects the secretion of growth hormones and insulin-like growth factor-I [16,17,18]. In other words, testosterone is closely associated with growth and muscle development. In this study, BW and ADG tended to be higher in the HH group than in the CH group (Table 2), and the size and diameter of LD muscle fibers increased in the HH group (Figure 2). Under these influences, carcass weight and rib eye area also increased (Table 3). Sundby et al. [19] reported that a higher serum testosterone concentration leads to greater BW and ADG, and Zhou et al. [20] observed higher expression of muscle-related genes in bulls than in steers. As a result, the CH group exhibited limited testosterone secretion after castration, but the HH group continued to secrete testosterone even after castration (Figure 1), which could be advantageous for growth and muscle development. In addition, testosterone concentrations increased up to 11 months of age in the HH group, which may be related to puberty and SSC. Nonghyup Agricultural Cooperative Federation Livestock Institute [21] reported that puberty occurs in Hanwoo cattle at approximately eight months and peak sexual maturity occurs at approximately 12 months, during which period the serum concentration of testosterone increases.

Cholesterol and triglyceride in the blood are included in the lipids absorbed in the small intestine or synthesized in the liver; thus, when the amount of feed intake increases, the concentration of cholesterol and triglyceride in the blood may increase [22]. In this study, although DMI was greater in the HH group, serum cholesterol and triglyceride concentrations were higher in the CH group for the duration of the test after surgical castration (Figure 1). Testosterone plays an important role in the regulation of cholesterol and triglyceride metabolism. For example, testosterone was recently reported to regulate the expression of hepatic proprotein convertase subtilisin/kexin type 9 and low-density lipoprotein receptor, as well as reduce cholesterol and triglyceride concentrations by affecting the activity of hepatic lipase [23,24]. Lee et al. [10] reported lower blood cholesterol and triglyceride concentrations in bulls than steers at 12, 18, 24, and 32 months of age. In other words, hemi-castration can regulate cholesterol and triglyceride metabolism in the liver, similar to that in bulls. Meanwhile, the decreased serum BUN concentration in the HH group may be due to anabolic effects. That is, the serum BUN levels are affected by the availability of ingested protein, and testosterone is an anabolic steroid that promotes nitrogen retention and improves protein synthesis [25], resulting in muscle growth and development [26]. In a previous study, testosterone treatment in pregnant ewes increased offspring N retention and decreased the serum BUN concentrations [27]. Of the two groups in this study, the serum BUN concentration was lower in the HH group, which had a high amount of testosterone secretion (Figure 1), the rib eye area increased (Table 3), and muscle development was evident (Figure 2). Therefore, hemi-castration can improve the metabolism and utilization efficiency of ingested proteins.

Adipose tissue, including intramuscular fat and subcutaneous fat, accumulates as the size of adipocytes and number of cells increase through the proliferation and differentiation of adipocytes. Adipocyte development begins in the cattle fetal period, and differentiation proceeds actively until the calf period [28]; however, their size increases through lipid synthesis rather than differentiation. In this process, various factors influence fat metabolism and development; however, testosterone is a representative inhibitory factor that interferes with the differentiation of adipocytes by decreasing the mRNA expression of peroxisome proliferator-activated receptor γ (PPARγ) and PPARγ activity [29] and reduces Glycerol-3-phosphate dehydrogerase activity in bovine intramuscular adipocytes to inhibit triglyceride synthesis [2]. In addition, as the testosterone concentration increases, the number of adipocytes and the expression of PPARγ are more severely reduced [16]. In this study, compared to the CH group, the size and deposition area of intramuscular adipocytes were decreased in the HH group, with the deposition area exhibiting a particularly large difference (Figure 2). This effect resulted in a reduction of marbling score in carcass evaluation (Table 3). We expected that the meat quality (marbling) of hemi-castrated cattle would be lower than that of steers but better than that of bulls. However, the carcass evaluation results of the HH group were similar to those of bulls slaughtered at a similar age. According to a KIAPQE [3] report presenting carcass evaluations of 970 bulls slaughtered at 24 months of age, the marbling score was 1.4 and the back fat thickness was 5.8. This could be explained by the abnormal development of one testicle. In a previous study, hemi-castration doubled the weight and diameter and increased the sperm count per testis from that of normal bull testes [30,31]. Therefore, the amount of testosterone secreted after hemi-castration may be equally sufficient for inhibiting the development of adipose tissue as that in bulls and may be more sensitive to differentiation than adipocyte hypertrophy.

Intramuscular fat, also known as marbling, is one of the most important meat quality traits affecting carcass grading and auction prices [32]. In a correlation analysis of the auction price of 762,749 Hanwoo cattle, the marbling score (r = 0.803, *p* < 0.0001) exhibited a strong positive linear relationship, and the carcass weight (r = 0.430, *p* < 0.0001) and rib eye area (r = 0.528, *p* < 0.0001) exhibited a moderate positive linear relationship. In contrast, meat color (r = −0.396, *p* < 0.0001) and texture (r = −0.617, *p* < 0.0001) exhibited a moderate negative linear relationship [3]. In this study, the marbling score of the HH group was 3.33 points lower than that of the CH group, whereas the meat color and texture were 0.99 and 1.71 points higher (Table 3), respectively. As a result of these effects, the auction price was 28.5% less for the HH group than the CH group, resulting in a significant difference in gross receipts and net income (Table 4). Although the carcass weight was higher in the HH group and the rib eye area was improved, it was not sufficient to overcome the difference in marbling score, suggesting that hemi-castration may be economically disadvantageous for high-quality beef production.

## 5. Conclusions

This study found that hemi-castration had some positive effect on growth performance and beef yield production through muscle development than castration due to the effect of testosterone secreted by one testis. However, by inhibiting the differentiation and lipid synthesis of adipocytes, hemi-castration also lowers the marbling score and auction prices to negatively affect net income. Thus, general castration, which removes both testicles, is essential in order to improve profitability through high quality beef production.

## Figures and Tables

**Table 1:** Ingredient and chemical composition of the experimental diets.

Item	Formula Feed		Roughage	
	Growing	Fattening	Italian Ryegrass	Rice Straw
	Ingredient composition (%)			
Corn grain	24.9	43.7		
Wheat grain	8.0	7.0		
Rice	-	2.0		
Cane molasses	3.0	3.6		
Wheat flour	-	1.5		
Wheat bran	6.9	3.0		
Corn gluten feed	21.0	11.0		
Soybean Meal	5.2	10.4		
Coconut meal	3.0	-		
Palm kernel meal	12.0	6.0		
Corn-DDGS ^1^	8.0	2.6		
Lupin flake	3.0	2.0		
Cottonseed	-	3.0		
Protected fat	-	0.4		
Salt dehydrate	0.6	0.7		
Limestone	2.9	1.9		
Sodium bicarbonate	0.3	0.7		
MSG-CMS ^2^	0.5	-		
Vitamin premix ^3^	0.1	0.1		
Mineral premix ^4^	0.1	0.1		
Feed additives	0.5	0.3		
	Chemical composition (%)			
Dry matter	88.06	86.97	84.64	91.64
Crude protein	16.02	14.50	10.38	4.73
Ether extract	4.37	4.38	2.79	2.29
Crude fiber	8.16	5.11	28.25	31.80
Crude ash	6.52	5.88	6.45	12.59
Neutral detergent fiber	29.32	17.45	56.09	63.19
Acid detergent fiber	13.29	7.56	31.20	36.78
TDN ^5^	72.00	75.00	-	-

^1^ Corn-DDGS: corn dried distill’s grains with solubles; ^2^ MSG-CMS: condensed molasses soluble-mono sodium glutamate; ^3^ Vitamin premix provided the following quantities of vitamins per kilogram of the diet: vitamin A = 10,000 IU, vitamin D_3_ = 1500 IU, vitamin E = 25 IU; ^4^ Mineral premix provided the following quantities of minerals per kilogram of the diet: Fe = 50 mg, Cu = 7 mg, Zn = 30 mg, Mn = 24 mg, I = 0.6 mg, Co = 0.15 mg, Se = 0.15 mg; ^5^ TDN, total digestible nutrients (calculated value).

**Table 2:** Comparison of growth performance on castrated and hemi-castrated Hanwoo.

Item	Treatment		*p*
	CH	HH	
Initial BW, kg	149.33 ± 19.46	147.53 ± 20.68	0.80
Final BW, kg	680.80 ± 64.72	716.00 ± 34.86	0.07
Average daily gain, kg	0.93 ± 0.11	1.00 ± 0.05	0.06
Dry matter intake, kg/d	8.43 ± 0.14 ^b^	8.53 ± 0.08 ^a^	0.02
Formula feed	6.37 ± 0.03 ^b^	6.44 ± 0.08 ^a^	0.01
Roughage	2.06 ± 0.12	2.09 ± 0.10	0.41
Feed conversion ratio	9.75 ± 1.56	9.10 ± 0.76	0.15

^a,b^ Means with difference superscripts in the same row are significantly different (*p* < 0.05).

**Table 3:** Comparison of carcass traits on castrated and hemi-castrated Hanwoo.

Item	Treatments		*p*
	CH	HH	
Yield trait			
Carcass weight (kg)	406.00 ± 50.21	427.78 ± 24.23	0.32
Rib eye area (cm^2^)	84.14 ± 5.43 ^b^	101.22 ± 11.19 ^a^	0.01
Back fat thickness (mm)	16.71 ± 2.69 ^a^	6.78 ± 2.28 ^b^	0.01
Yield index	60.26 ± 0.94 ^b^	67.58 ± 1.30 ^a^	0.01
Yield grade score ^1^	1.57 ± 0.53	2.11 ± 0.78	0.12
Quality trait			
Marbling score	5.00 ± 1.53 ^a^	1.67 ± 1.32 ^b^	0.01
Meat color	4.57 ± 0.79 ^b^	5.56 ± 0.73 ^a^	0.02
Fat color	3.14 ± 0.38	3.33 ± 0.50	0.40
Texture	2.29 ± 0.95 ^b^	4.00 ± 1.12 ^a^	0.01
Maturity	1.86 ± 0.38	2.22 ± 0.44	0.10
Quality grade score ^2^	3.29 ± 1.11 ^a^	1.44 ± 0.73 ^b^	0.01
Auction price (USD ^3^/kg)	16.88 ± 2.15 ^a^	12.08 ± 2.48 ^b^	0.01

^a,b^ Means with difference superscripts in the same row are significantly different (*p* < 0.05). ^1^ Yield grade score: Grade A = 3, grade B = 2, and grade C = 1; ^2^ Quality grade score: Grade 1^++^ = 5, grade 1^+^ = 4, grade 1 = 3, grade 2 = 2, and grade 3 = 1; ^3^ USD: United States dollar.

**Table 4:** Comparison of economy efficacy on castrated and hemi-castrated Hanwoo.

Item	Treatments		*p*
	CH	HH	
USD ^1^/head			
Gross receipts ^2^ (A)	6923.9 ± 1591.4 ^a^	5186.2 ± 1212.0 ^b^	0.04
Operating costs (B)	5247.3 ± 27.2 ^b^	5269.2 ± 15.7 ^a^	0.01
Calf ^3^	3409.1	3409.1	-
Feed costs ^4^	1838.3 ± 27.2 ^b^	1860.1 ± 15.7 ^a^	0.01
Commercial concentrate	1458.5 ± 7.9 ^b^	1474.4 ± 19.6 ^a^	0.01
Growing formula feed	517.0 ± 2.7	517.3 ± 4.7	0.85
Fattening formula feed	941.5 ± 7.3 ^b^	957.1 ± 17.0 ^a^	0.01
Roughage	379.8 ± 22.2	385.8 ± 19.4	0.43
Italian ryegrass	284.1 ± 16.8	288.3 ± 15.5	0.47
Rice straw	95.7 ± 5.8	97.5 ± 4.2	0.33
Net income (C = A-B)	1677.1 ± 1586.9 ^a^	−82.9 ± 1212.8 ^b^	0.04

^a,b^ Means with difference superscript in the same row are significantly different (*p* < 0.05). ^1^ USD: United States dollar; ^2^ Gross receipts: Selling price of carcass and by product; ^3^ Calf: average auction price of Hanwoo calf in 2020; ^4^ Feed costs: Commercial concentrate (growing formula feed = 0.35 USD/kg + fattening formula feed = 0.36 USD/kg) + roughage (italian ryegrass 0.31 USD/kg + rice straw: 0.22 USD/kg).

## Data Availability

Not applicable.
